# Integrated One Health Surveillance of West Nile Virus and Usutu Virus in the Veneto Region, Northeastern Italy, from 2022 to 2023

**DOI:** 10.3390/pathogens14030227

**Published:** 2025-02-25

**Authors:** Federica Gobbo, Giulia Chiarello, Sofia Sgubin, Federica Toniolo, Francesco Gradoni, Lidia Iustina Danca, Sara Carlin, Katia Capello, Giacomo De Conti, Alessio Bortolami, Maria Varotto, Laura Favero, Michele Brichese, Francesca Russo, Franco Mutinelli, Stefania Vogiatzis, Monia Pacenti, Luisa Barzon, Fabrizio Montarsi

**Affiliations:** 1 Istituto Zooprofilattico Sperimentale delle Venezie, Viale dell’Università 10, 35020 Legnaro, Italy; gchiarello@izsvenezie.it (G.C.); ssgubin@izsvenezie.it (S.S.); ftoniolo@izsvenezie.it (F.T.); fgradoni@izsvenezie.it (F.G.); ldanca@izsvenezie.it (L.I.D.); scarlin@izsvenezie.it (S.C.); kcapello@izsvenezie.it (K.C.); gdeconti@izsvenezie.it (G.D.C.); abortolami@izsvenezie.it (A.B.); mvarotto@izsvenezie.it (M.V.); fmutinelli@izsvenezie.it (F.M.); fmontarsi@izsvenezie.it (F.M.); 2 Department of Prevention, Food Safety and Veterinary, Veneto Region, 30123 Venice, Italy; laura.favero@regione.veneto.it (L.F.); michele.brichese@regione.veneto.it (M.B.); francesca.russo@regione.veneto.it (F.R.); 3 Department of Molecular Medicine, University of Padua, Via A. Gabelli 63, 35121 Padova, Italy; stefania.vogiatzis@unipd.it (S.V.); monia.pacenti@aopd.veneto.it (M.P.); luisa.barzon@unipd.it (L.B.)

**Keywords:** West Nile virus, Usutu virus, Marisma virus, One Health, arbovirus, orthoflavivirus, surveillance, mosquito, bird, human encephalitis

## Abstract

West Nile virus (WNV) and Usutu virus (USUV) are neurotropic mosquito-borne orthoflaviviruses maintained in an enzootic cycle, in which birds are amplifying/reservoir hosts, while humans and equids are dead-end hosts. As northern Italy, especially the Veneto Region, is considered an endemic area for WNV and USUV circulation, a surveillance plan based on a One Health approach has been implemented since 2008. This work reports the results of entomological, veterinary and human surveillances for WNV and USUV in the Veneto Region in 2022 and 2023, through virological and/or serological examinations. In 2022, 531 human WNV infections were recorded, and 93,213 mosquitoes and 2193 birds were virologically tested, showing infection rates (IRs) of 4.85% and 8.30%, respectively. The surveillance effort in 2023 provided these results: 56 human WNV infections were confirmed, and 133,648 mosquitoes and 1812 birds were virologically tested, showing IRs of 1.78% and 4.69%, respectively. This work highlights the exceptional circulation of WNV in the Veneto Region, due to the new re-introduction of WNV lineage 1 and co-circulation with WNV lineage 2. This paper confirms the efficacy of integrated surveillance for early warning of viral circulation and gives new insights about avian hosts involved in the enzootic cycle of orthoflavivirus in the endemic region of Italy.

## 1. Introduction

Vector-borne infectious diseases represent a significant and growing issue in the European Union. Mosquito-borne viral infectious diseases such as West Nile disease (WND) and Usutu-related disease are endemic in many European countries; West Nile virus (WNV) has been circulating in Mediterranean countries at least since the 1960s [1] and Usutu virus (USUV) at least since 1996 [2]. Recently, local infections in the animal reservoir and in humans have also been reported in the northern part of EU, like in Germany, and in the Netherlands [3,4,5,6].

WNV and USUV are RNA viruses belonging to the genus *Orthoflavivirus* (family *Flaviviridae*) [7]. These viruses are transmitted by mosquito vectors between wild and domestic birds, which act as amplifying/reservoir hosts. Humans and equids are incidentally infected through mosquito bite and may develop severe neurological disease, but they are dead-end hosts because the low level of viremia cannot sustain onward transmission [8,9].

WNV has been repeatedly introduced in Europe by migratory birds from Africa in southern and western Europe, while European-resident birds may maintain the virus during overwintering [10,11,12,13]. Out of the nine lineages of WNV identified so far, six have been found in Europe (lineages 1, 2, 3, 4, 8 and 9). However, only WNV lineage 1 (WNV-1) and WNV-2 have been associated with disease in humans, with WNV-1 being predominant until 2004, when WNV-2 sub-lineage 2a emerged in Hungary and then spread to southern and western European countries causing large human outbreaks [14]. In Europe, WNV is transmitted by mosquito species of the genus *Culex*, mainly *Culex pipiens* and *Cx. modestus*; *Aedes species* (i.e., *Aedes albopictus* and *Ae. caspius*) have been found infected but they have a minor role for the transmission [15].

Several avian species have been confirmed susceptible to WNV infection in Europe and clinical signs and mortality have been reported mainly in birds of prey (orders Accipitriformes, Falconiformes and Strigiformes) and passerine birds [4,16,17,18,19].

Infection in humans is usually asymptomatic; 20–30% of infected subjects develop a mild influenza-like syndrome, defined as West Nile fever (WNF), while less than 1%, mainly elderly and immunocompromised individuals, develop a neuro-invasive disease (WNND), with encephalitis, meningitis, and/or acute flaccid paralysis. In patients with WNND, mortality is approximately 10–20% and severe sequelae persist in 20–30% of those who survive [20].

In Italy, the regions reporting most human cases of WNV infection are Veneto, Lombardy, Emilia Romagna, Piedmont and Sardinia. With the exception of Sardinia, an island in the Tyrrhenian Sea, all the mentioned regions are located in the Po Valley, which mainly consists of plain landscape with wide wetland areas that serves as stopover and wintering sites for many wild waterfowl migrating across southern, northern and eastern Europe [21].

Like WNV, USUV has an enzootic cycle between mosquitoes and birds with humans, bats and other mammals as dead-end hosts [22]. After its introduction in Europe, USUV caused massive mortality of wild avifauna, mostly among Eurasian Blackbirds (*Turdus merula*) [23,24,25] and birds of prey in captivity [26]. Eight USUV lineages have been identified and those detected in Europe are the lineages Europe 1, 2, 3 and 4 and the lineages Africa 2 and 3 [27]. In Italy, at least four lineages (EU1–4) have been circulating among mosquitoes and wild birds [28]. EU2 circulates in Emilia Romagna, Veneto and Friuli-Venezia-Giulia Regions, whereas EU4 mainly circulates in Piedmont Region [29]. Most USUV infections in humans are asymptomatic and incidentally detected during screening of blood donors for WNV infection with nucleic acid amplification tests that cross-react with USUV [30]. Although rare, neuro-invasive USUV infections in humans have been reported across several European countries, such as Croatia, Czech Republic, Hungary, Switzerland, Austria, France and Italy [27,31,32].

Italy is the European country with the highest circulation of WNV and USUV and consequently the highest number of cases of infection in humans and animals [33]. In 2010, the first National WNV Surveillance Plan, mandated by the Italian Ministry of Health, was based on an integrated One Health surveillance approach (veterinary, entomological and human surveillance) [34]. In 2020, the Italian Ministry of Health combined the surveillance plans for different arboviruses into the “National Plan for Prevention, Surveillance and Response to Arboviruses 2020–2025” (PNA 2020–2025) [35]; this plan enhanced surveillance and early warning of vector-borne viral infections, communication, safety measures for Substances of Human Origin (SoHO) donors, and vector control measures in a One Health perspective.

The aim of the present study is to report the results of the integrated surveillance for WNV and USUV (2022 and 2023) through the application of the PNA 2020–2025 in the Veneto Region, which is considered an endemic area for both orthoflavivirus. It is worth mentioning that in 2022, the Veneto Region reported a large WNV epidemic with an extraordinarily high number of human cases of infection compared to other EU countries [33,36].

## 2. Materials and Methods

### 2.1. Background

In Italy, the PNA 2020–2025 regulates the surveillance of WNV and USUV and defines aims and manners of activities relating to entomological, veterinary and human surveillances. According to the Plan, entomological surveillance is carried out during the period of mosquito activity, from May to October, while veterinary and human surveillance last throughout the year. Specifically, this work reports the surveillance activities carried out during 2022 and 2023 in the provinces of the Veneto Region at high risk of transmission: Padua, Rovigo, Treviso, Venice, Vicenza, and Verona.

### 2.2. Entomological Surveillance

**Figure 1:**
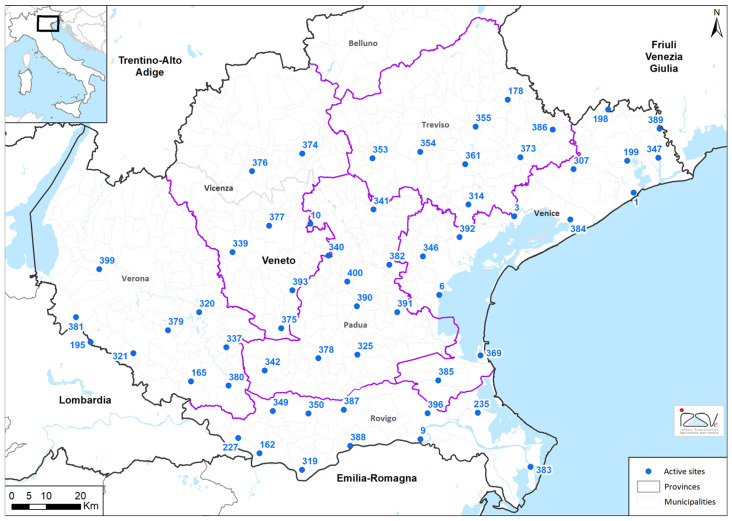
Geographical distribution of collection sites in the Veneto Region. Purple line: provinces of the Veneto Region; Blue number dots: traps identification number (trap ID).

Entomological surveillance consisted of bi-weekly samplings using CDC-like traps (Centre for Disease Control and Prevention-like trap; Italian Mosquito Trap IMT^®^; PeP, Cantu, Italy) baited with dry ice pellets as source of CO_2_ (CDC-CO_2_) and homemade Gravid Trap-like (similar to model by John W. Hock Company^©^, Gainesville, FL, USA, CDC Gravid trap-model 1712), both activated for one night in each selected collection site for the whole surveillance season. Thus, in 2022 and 2023, surveillance activities were carried out from mid-May to mid-October using 57 CDC traps and 7 Gravid traps. Traps were geographically distributed all over the plan area of the Veneto Region (below 300 m above the sea level and approx. one trap every 120 km^2^) and geo-referenced (Figure 1; Supplementary Table S1). CDC-CO_2_ traps were strategically allocated in rural environments to enhance the capture of *Cx. pipiens*, while Gravid traps were used in urban/periurban sites [37]. Collected mosquitoes were labelled and stored at 4 °C during the delivery to the laboratory. On arrival, collected mosquitoes were counted and identified under stereomicroscope according to taxonomic keys [38]. Females belonging to the *Cx. pipiens* complex are reported as *Cx. pipiens* encompassing *Cx. pipiens pipiens*/*molestus*, *Cx. perexiguus* and *Cx. torrentium*, *Ae. albopictus* and *Ae. caspius* were divided into pools of maximum 100 specimens according to day of collection, species and capture site, and then submitted for orthoflavivirus screening, whereas all other collected mosquitoes’ species were only counted, identified and stored at −20 °C. The molecular screening was performed using an in-house developed one-step SYBR green-based real-time RT-PCR assay followed by virus identification by Sanger sequencing, as previously described by Ravagnan and colleagues [39]. All WNV- and/or USUV-positive samples were sent to the National Reference Centre for Exotic Diseases of Animals (CESME) for laboratory confirmation as prescribed by the PNA 2020–2025.

### 2.3. Veterinary Surveillance

Veterinary activities consisted of a passive surveillance in wild avifauna, which was carried out during the entire year, and an active surveillance in the spring and summer seasons in birds considered target species for WNV and USUV infections such as Eurasian Magpie (*Pica pica* Linnaeus, 1758), Hooded Crow (*Corvus cornix* Linnaeus, 1758), Carrion Crow (*Corvus corone* Linnaeus, 1758) and Eurasian Jay (*Garrulus glandarius* Linnaeus, 1758). From each bird the brain, heart, spleen and kidney were sampled and pooled together for WNV-1, WNV-2 and USUV real-time RT-PCR screening, as previously described [40]. Since 2016, equids (horses and donkeys) have been subject to syndromic surveillance. Hence, in the case of clinical symptoms correlated with WNV infection, the official veterinarians collected samples from the affected animals. Diagnostic samples consisted of blood serum for serological testing performed using a commercial ELISA kit for the detection of IgM versus WNV (ID Screen West Nile IgM Capture; IDVet^®^, Grabels, France) and blood in K3EDTA for the diagnosis of WNV viremia by real-time RT-PCR as previously described [41]. Screening for USUV was not provided for equids. All animal positive samples for WNV and USUV were sent to the CESME for laboratory confirmation.

### 2.4. Human Surveillance

Case definition of WNV and USUV infection was according to PNA 2020–2025. Individuals with neurological symptoms or with febrile illness were tested as possible cases of human infection. Confirmed cases were defined as individuals (with or without symptoms) presenting with at least one of the following laboratory criteria: virus isolation in cell culture monolayer (VERO) from serum, urine and/or cerebrospinal fluid (CSF); molecular detection of viral RNA in blood, urine and/or CSF; detection of a specific IgM antibody response in CSF; high IgM antibody titer and detection of IgG antibodies in serum and confirmation by neutralization assays. A probable case was defined as individuals with fever or neurological symptoms and IgM antibodies detected in serum, without confirmation with a neutralization assays. Laboratory investigation of suspected WNV infections was carried out as previously described [36].

## 3. Statistical Analysis

For mosquitoes, the infection rate (IR) was defined as the number of mosquito pools testing positive divided by the total number of pools tested; for birds, IR was defined as the number of birds testing positive divided by the total number of birds tested; 95% confidence intervals were provided. A two-sample test of proportions was used to evaluate statistical differences in IRs between the two years. Statistical evaluation of the associations between categorical factors was performed using Pearson’s χ^2^ test or Fisher’s exact test. The same statistical tests were used also for avifauna data; notwithstanding, in the evaluation of orders, the statistical analysis was performed only for those with at least 10 positive samples recorded over the entire period. Results were considered statistically significant with a *p*-value (*p*) ≤ 0.05. Software STATA v.17.0 was adopted (StataCorp. 2021. Stata: Release 17. Statistical Software. College Station, TX, USA: StataCorp LLC).

## 4. Results

### 4.1. Entomological Surveillance

A total of 93,213 and 133,648 mosquitoes were collected in 2022 and 2023, respectively (Table 1), belonging to 16 species of *Culicidae*. The predominant species was *Cx. pipiens* (58.94% in 2022 and 82.90% in 2023), the vector targeted by the WNV surveillance plan, followed by *Ae. caspius* (30.14% in 2022 and 8.59% in 2023) and *Ae. albopictus* (4.46% in 2022 and 3.58% in 2023). For 4757 mosquitoes (5.10%) in 2022 and 4913 (3.68%) in 2023, the morphological identification was not possible due to damaged specimens. The CDC-CO_2_ trap collected 98.1% of the total mosquitoes (aggregated data 2022–2023).

Pools containing a total of 2123 and 2301 target mosquitos were tested for orthoflaviviruses in 2022 and 2023, respectively, and the percentages found to be positive for each province are reported in Table 2 and Table 3.

Interestingly, in 2022, several pools presented an unexpected rate of viral co-infections (Table 2) and one pool of 58 *Cx. pipiens* collected in the Province of Padua on August 1 showed a triple infection with WNV-1, WNV-2 and USUV (trap ID 342) (Supplementary Table S2a).

**Figure 2:**
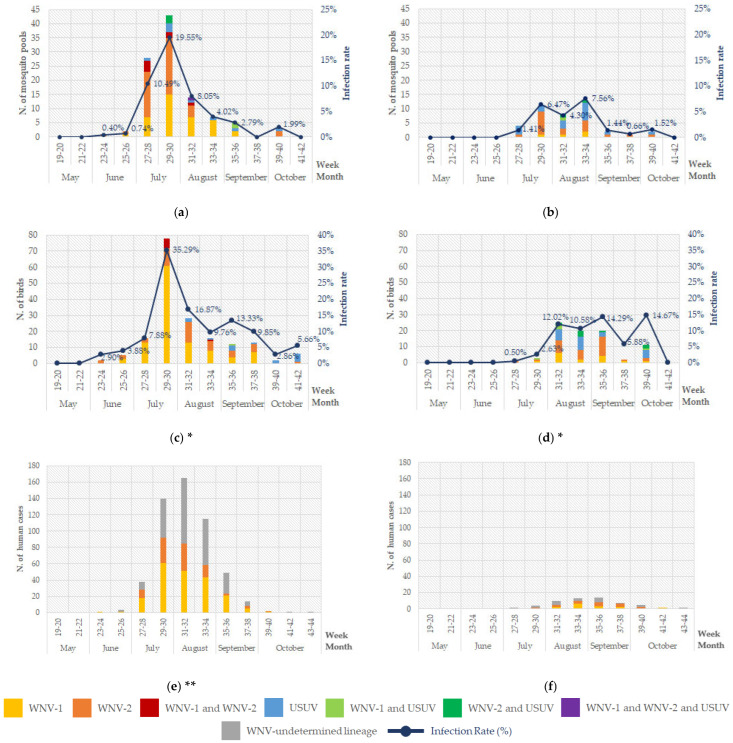
Positive results of the integrated surveillance in the Veneto Region: (**a**) Entomological surveillance in 2022; (**b**) entomological surveillance in 2023; (**c**) avifauna surveillance in 2022; (**d**) avifauna surveillance in 2023; (**e**) human surveillance in 2022; (**f**) human surveillance in 2023. WNV-undetermined lineage: by the means of virological and/or serological examinations. *: thirty-four birds and twenty birds are excluded in 2022 and 2023, respectively, because of the lack of date details. **: two confirmed human infections are excluded, because of the lack of date details.

In summer 2022, an earlier WNV circulation than in the previous years was detected in mosquitoes, with the first positivity for WNV-2 noted on June 6 in a pool of 56 *Cx. pipiens* (trap ID 393) in the Province of Vicenza. The first evidence of WNV-1 was noted in the Province of Venice in a pool of 46 *Cx. pipiens* on June 20 (trap ID 385) (Supplementary Table S2a). In the following months, WNV-1, WNV-2 and USUV were detected throughout the region, showing high IRs from July to August 2022, with a peak of IR (19.55%) in late July (weeks 29–30), followed by its drastic decline in late August (Figure 2a). Early and high IRs of WNV-1 and WNV-2 were found in the Provinces of Padua, Rovigo and Venice with peaks of IR in weeks 29–30 (Padua 38.10%, Rovigo 44.64%, and Venice 30.68%). In the Provinces of Verona and Vicenza (western Veneto Region), WNV-positive mosquito pools were detected later and for a longer period, in fact the highest IR values were recorded in weeks 30–31 (Verona 23.53% and Vicenza 10.00%) (Supplementary Figure S1).

Regarding USUV, the findings in 2022 were surprising because it was poorly detected in mosquitoes at the beginning of the surveillance season (Figure 2a) and few positive pools were collected in July in all provinces except the Province of Rovigo, where the circulation was demonstrated later in mid-October (Supplementary Figure S1).

Positive pools for WNV and/or USUV were mainly those of *Cx. pipiens* (98 pools out of 108, 90.74%); however, three pool of *Ae. albopictus* (2.78%) collected in July and August 2022 were positive for WNV-1 and WNV-2 in the Province of Venice, and two pools of *Ae. caspius* (1.85%) collected in August and October 2022 were positive for other WNV-2 in the Province of Venice. Another five pools (4.63%) of *Ae. caspius* were found positive for others orthoflaviviruses, such as Marisma virus (*n* = 4) and Mosquito Flavivirus (*n* = 1) in Provinces of Venice and Rovigo (Supplementary Table S2a).

In 2023, orthoflavivirus detection in vectors showed a trend similar to other years before 2022, observing from the end of June and the beginning of July the initial circulation of Marisma virus (June 27) and other Mosquito Flaviviruses (July 11) in *Ae. caspius* and USUV in *Cx. pipiens* (July 6) (Supplementary Table S2b). The temporal distribution of orthoflavivirus-positive mosquito pools and the relative IR are shown in Figure 2b. The first WNV-2 positivity was noted on July 12 in a pool of 100 *Cx. pipiens* from the Verona Province (trap ID 320), whereas the first WNV-1 positivity was noted on July 17 in a pool of 100 *Cx. pipiens* from the Rovigo Province (trap ID 396) (Supplementary Table S2b). The temporal distribution of orthoflavivirus-positive mosquito pools in each province are available in the Supplementary Figure S2.

In 2023, USUV-positive pools were found during the summer period until August in all provinces, except in the Province of Vicenza, where positive pools were found also in September and October (Supplementary Figure S2). WNV and/or USUV-positive pools were mainly *Cx. pipiens* (39 out of 47 orthoflavivirus-positive pools, 82.98%), *Ae. albopictus* (2 out of 47 orthoflavivirus-positive pools, 4.26%) collected in the Province of Padua and Verona. On the contrary, six *Ae. caspius* pools (12.77%) collected in the Provinces of Padua, Venice, Vicenza and Rovigo were positive for mosquito-only orthoflaviviruses, such as Marisma virus (*n* = 2) and Mosquito Flavivirus (*n* = 4) (Supplementary Table S2b).

Temporal distribution of WNV and USUV-positive mosquito pools and IR in 2022 and 2023 in the Veneto Region are shown in Figure 2a,b. Additionally, temporal distribution of mosquito positivity and IR for each province of the Veneto Region (Padua, Rovigo, Treviso, Venice, Verona, and Vicenza) are available in Supplementary Figure S1 for 2022 and Figure S2 for 2023, respectively.

In 2022, the highest number of positive pools were found in the Rovigo (*n* = 30) and Venice (*n* = 31) Provinces (Table 2), while in 2023, the highest number of positive pools were detected in Venice (*n* = 14) (Table 3), but no significance differences of Irs were observed among the different provinces of the Veneto Region for both years. However, a significant higher IR in 2023 was highlighted compared to 2022 (*p* < 0.001). In fact, the Irs were 4.85% (*n* = 103, 95% CI: 3.97–5.85) and 1.78% (*n* = 41, 95% CI: 1.28–2.41) for 2022 and 2023, respectively. This result was attributable to the significant decrease (*p* < 0.001) in positivity for WNV. The Irs for WNV-1 and WNV-2 were, respectively, 2.26% (95% CI: 1.63–2.89%) and 2.59% (95% CI: 1.91–3.26%) in 2022 compared to 0.22% (95% CI: 0.03–0.41%) and 0.87% (95% CI: 0.48–1.24%) in 2023; no significant difference was observed in the Irs for USUV (IR = 0.66% 95% CI: 3.15–1.00% in 2022 and IR = 0.83% 95% CI: 4.56–1.19% for 2023). Additionally, the distribution of positive pools among the different virus types highlighted that the decrease in WNV positivity was specifically driven by a reduction in WNV-1: the percentage of positive pools for WNV-1 among all positives pools decreased from 46.60% in 2022 to 12.20% in 2023, while for WNV-2, it remained relatively stable at 53.40% in 2022 and 48.78% in 2023.

### 4.2. Veterinary Surveillance

*Avifauna*: in 2022, a total of 2193 birds were tested for orthoflaviviruses (Table 4), with Passeriformes being the most represented taxonomic order (42.95%). Most birds were collected in the Venice (*n* = 557, 25.40%) and Verona (*n* = 571, 26.04%) Provinces. In 2023, a total of 1812 birds were tested, of which 38.69% were Passeriformes (Table 4).

In summer 2022, an unexpected and earlier WNV circulation was also detected in wild birds (Figure 2c). A total of 182 birds out of 2193 were positive for WNV and/or USUV (8.30%) in the year 2022, as detailed in Table 5.

The first positive bird for WNV-2 was a Common Kestrel (*Falco tinnunculus*), found in the Padua Province on 7 June 2022. Evidence of WNV-1 circulation in avifauna was also detected in late June in a Rook (*Corvus* spp.) in the Venice Province, in a Little Owl (*Athena noctua*) in the Padua Province and later in a Sparrow (*Passer* spp.) in the Rovigo Province.

Both WNV lineages spread in the wild avifauna of the Veneto Region with a peak of IR (35.29%) in weeks 29–30, as observed for mosquitoes (Figure 2a,c). USUV-positive birds were found from August to late-October, mostly in Passeriformes, in particular in Common Blackbirds (*Turdus merula*). Overall, 111 birds (5.06%) belonging to 11 taxonomic orders were WNV-1-positive as a single infection and 47 birds (2.14%) of 6 taxonomic orders were WNV-2-positive as a single infection. Co-infection with both WNV lineages were detected in seven (0.32%) birds belonging to 4 orders. Finally, USUV was detected as a single viral infection in 16 birds (0.73%) of 5 orders; one Common Wood Pigeon (*Columba palumbus*) presented USUV and WNV-1 co-infection (Table 4).

Temporal distribution of wild positive birds in 2022 and the relative infection prevalence in each province are available in the Supplementary Figure S3.

In summer 2023, a total of 85 birds out of 1812 (4.69%) were positive for WNV and/or USUV. Detailed information of positive birds are available in Table 6.

As for the mosquitoes, orthoflavivirus detection in wild birds showed a trend similar to other years before 2023 (Figure 2b,d). The first positivity for WNV-2 was noted in a Common Wood Pigeon (*Columba palumbus*), found in the Venice Province on 3 July 2023. The first evidence of WNV-1 circulation in avifauna was registered later in a Grey Heron (*Ardea cinerea*) also co-infected with USUV, rescued in the Venice Province on 28 July 2023. Both lineages of WNV and USUV were widespread in wild avifauna of the Veneto Region, showing an average IR of 11.49% from August to mid-October 2023 (Figure 2d).

Specifically, 16 birds (0.89%) belonging to 4 taxonomic orders had WNV-1 infection; 32 birds (1.79%) of 7 taxonomic orders had WNV-2 infection; 24 birds (1.34%) of 5 taxonomic orders had USUV infection. No co-infections with both lineage of WNV were detected in any tested birds, whereas USUV co-infections with WNV-1 or WNV-2 were evidenced in four birds (orders Anseriformes, Columbiformes, Passeriformes, and Pelecaniformes) and nine birds belonging to order Columbiformes, respectively (Table 6).

Temporal distribution of positive birds in 2023 and IR for each province of the Veneto Region are reported in the Supplementary Figure S4.

A significantly higher rate of positive birds for any orthoflavivirus was observed in 2022 compared to 2023: 2022, IR = 8.29% (95% CI: 7.17–9.53); 2023, IR = 4.69% (95% CI: 3.76–5.76). A decrease in IR was recorded in 2023 compared to 2022 for Charadriiformes (*p* = 0.010), Passeriformes (*p* = 0.001), and Strigiformes (*p* < 0.001). For the latter, the IR dropped from 20.88% to 3.23%. No significant differences were statistically observed in the IR for Columbiformes and Pelecaniformes (Table S3a). Examining the distribution of positive samples by order and year, a significant difference was found (*p* < 0.001). In 2022, 50.30% of the positive samples were from Passeriformes, followed by 23.60% from Strigiformes. In contrast, in 2023, Columbiformes accounted for 46.25% of all positive samples, followed by 40.00% from Passeriformes.

A significant association between the positivity to orthoflavivirus and migratory patterns was observed in both years (2022: *p* = 0.019; 2023: *p* = 0.001). The highest percentage of positives avian samples were found in the residential birds in 2022 (10.40%) and in the migratory birds in 2023 (6.34%) (Table S3b).

*Equids*: in 2022, WNV infection was detected in horses reared in the Padua Province (*n* = 6), Treviso (*n* = 2), Vicenza (*n* = 1), and Venice (*n* = 1). The first cases were registered in the Padua Province on July 12 demonstrating an earlier infection also in equids. In 2023, four suspected cases of WNV infection were reported but not confirmed by the National reference laboratory CESME.

### 4.3. Human Surveillance

In 2022, a total of 531 human WNV infections were confirmed by the Veneto Region Reference Laboratory. Although in 43.69% of human cases (*n* = 232) the attribution of the viral lineage was not determined, most human cases had WNV-1 infection and the provinces with the highest number of cases were Padua and Venice (Table 7). The first human case was from the Padua Province reporting the onset of symptoms on 18 June 2022 (week 24), approximately two weeks later the first WNV detection in vectors.

No human cases of USUV Infection were Identified. However, In eight patients (six from the Padua, one from Vicenza, and one from Verona Provinces), a high neutralizing antibody titer against both WNV and USUV was demonstrated and thus it was not possible to determine which of two viruses was the causative agent of the current infection (these eight cases are not included in Table 7 and Figure 2e).

In 2023, 56 human WNV infections were reported with the attribution of the viral lineage in half of cases (in 23 cases WNV lineage was not determined); most infections were reported in the Provinces of Padua and Verona (Table 7). The first human case in 2023 was noted on July 5 (week 27) in the Padua Province, a few days after the first detection of WNV in a Common Wood Pigeon. The temporal distribution of WNV in humans for 2022 and 2023 is shown in Figure 2e and Figure 2f, respectively.

Overall, human WNV infections recorded in 2023 were ten times lower than in 2022 (2022: *n* = 531; 2023: *n* = 56), whereas for those human infections in which the lineage was determined, a significantly different distribution was observed between the two years (*p* = 0.011), principally due to WNV-1: the percentage of cases decreased from 38.23% in 2022 to 25.00% in 2023. Conversely, WNV-2 cases increased of 15.85 percentage points (Table 7 and Table S3c).

**Figure 3:**
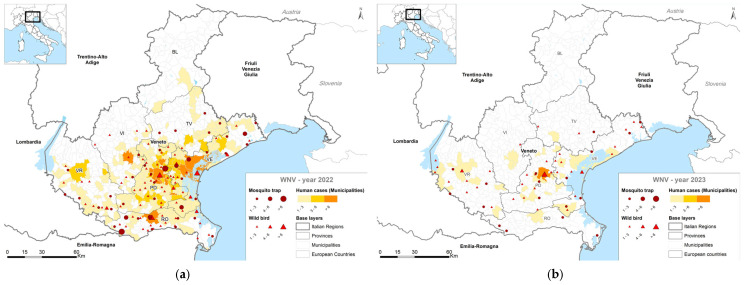
Geographical distribution of WNV infections in mosquitoes, wild birds and humans of the Veneto Region in (**a**) 2022; (**b**) 2023. BL: Belluno Province; PD: Padua Province; RO: Rovigo Province; VE: Venice Province; VR: Verona Province; VI: Vicenza Province. Red dots: positive mosquito trap site; Red triangle: positive wild birds; Yellow to orange shades: positive human cases.

The geographical distribution of WNV infections in humans, birds, equids and mosquitoes for the 2022 and 2023 are reported in Figure 3a,b. The maps show that the greatest circulation of WNV in 2022 was in the Provinces of Padua, Venice and Rovigo, while in 2023, the distribution of the virus across the territory was more homogeneous.

**Figure 4:**
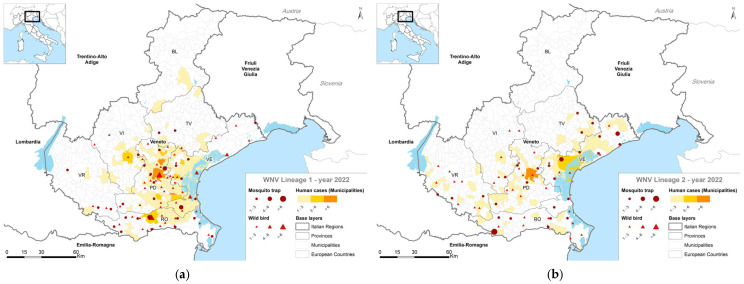

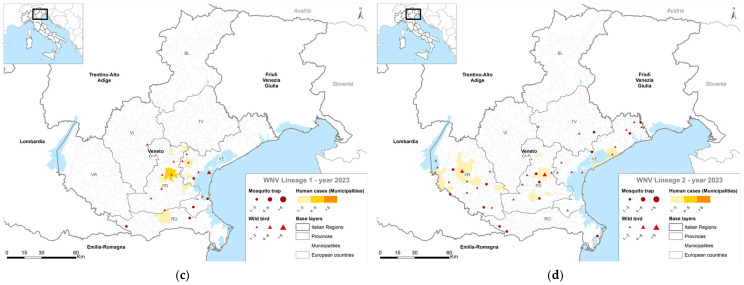
Geographical distribution of WNV-1 or WNV-2 infections in mosquitoes, wild birds and humans of the Veneto Region: (**a**) WNV-1 in 2022; (**b**) WNV-2 in 2022; (**c**) WNV-1 in 2023; (**d**) WNV-2 in 2023. BL: Belluno Province; PD: Padua Province; RO: Rovigo Province; VE: Venice Province; VR: Verona Province; VI: Vicenza Province. Red dots: positive mosquito trap site; Red triangle: positive wild birds; Yellow to orange shades: positive human cases.

Considering the geographical distribution of WNV-1 and WNV-2 in humans, birds and mosquitoes (Figure 4), the maps show that the overall circulation of WNV was mainly attributed to an extensive circulation of WNV-1 lineage in the Provinces of Padua, Venice and Rovigo in 2022.

## 5. Discussion

In the Veneto Region, the WNV transmission season in 2022 was characterized by an early circulation of the virus in the vector *Cx. pipiens* and consequently an earlier viral infection in wild birds, and precocious occurrence of a high number of cases of WNV infection (WNF and WNND) in mammal dead-hosts such as equids and humans. These findings suggest unexpected and high viral pressure in the enzootic cycle of WNV. It is worth remembering that in 2022, in addition to the endemic WNV-2, the newly introduced WNV-1 (detected for the first time in Veneto in August 2021 [42]) had a significantly higher rate of infection in mosquitoes, birds and human and was the major contributor of the epidemic behavior in 2022 in Italy and specifically in the Veneto Region. Moreover, most WNND and fatalities in humans were attributable to WNV-1, suggesting increased virulence [36]. Phylogenetic analysis of the newly introduced WNV-1 classified this viral strain within Clade 1A, of the Mediterranean subtype, and it showed high similarity with two viral strains detected in France in 2015 and 2018 [42], thus it is possible to speculate that its introduction in northern Italy was related to migratory birds and their movements along migratory routes in the Mediterranean basin [43].

After its first introduction in Italy in 1998 [44], WNV-1 was sporadically reported until 2008, when significant circulation resulted in human outbreaks between 2008 and 2011 especially in northern Italy and the Sardinia Region [45]. The WNV-1 was replaced in the following years by WNV-2, which became the dominant lineage both in Italy [46], and in other European countries [14]. In 2020, WNV-1 was sporadically detected in the Campania Region, a southern region of Italy, where WNV circulation was not recorded in previous years [47] followed by more frequent detections in 2021 in humans, mosquito vectors and birds of the Veneto Region [42]. WNV-1 detections then occurred across the entire Veneto Region in 2022, showing high IRs in wild avifauna [36], suggesting a lack of competent immunity in avian populations of residential birds.

This hypothesis is supported by the evidence of a significant association between orthoflavivirus positivity in avifauna and migratory patterns: the year 2022 showed that birds displaying a residential pattern were associated with the highest rate of positivity (10.40%) while in 2023, the highest IR was observed in birds associated with a migratory pattern (6.34%) (Table S3b).

Where available, the clinical history of WNV-1 infections in horses or birds (mainly birds of prey) reported severe neurological disorders in affected animals according to previous findings [16]. In 2023, WNV circulation decreased as evidenced by fewer positives in mosquitoes and this was specifically driven by a reduction in WNV-1 IR, while for WNV-2, it remained relatively stable. Recent phylogeographic analysis of the Italian genome of WNV-2 demonstrated the circulation of four different clades (1–4) in Italy; and at the subregional level, Clades 1 and 4 are those reported in the Veneto Region; the former emerged around 2013–2014 and then disappeared, whereas the latter is the predominant clade involved in the epidemics in 2018 and 2022 in Italy [46].

According to data obtained from active and passive surveillance of wild birds, the main taxonomic orders with species susceptible to natural infection with WNV were Strigiformes, Passeriformes, Falconiformes, Accipitriformes, Anseriformes, Charadriiformes, Pelicaniformes and Psittaciformes as already described in the literature [18,19,48,49]. Interestingly, we detected WNV-1 and WNV-2 in single or multiple infection in additional Orders: Apodiformes (*Apus apus*), Columbiformes (*Columba palumbus*, *Streptopelia* spp. and *Columba livia*), Piciformes and Pelecaniformes (*Phalacrocorax carbo*).

Regarding USUV infection in wild avifauna, our data confirm natural infection in birds of orders Passeriformes (mainly *Turdus merula*), Strigiformes, Columbiformes (*Columba palumba*, *Streptopelia* spp. and *Columba livia*), Pelicaniformes (*Ardea cinerea* and *Ardeola ibis*) [18,19,50] and Anseriformes (*Anas platyrhynchos* and *Mareca strepera*). Thus, this study gives new insight about avian hosts that can be involved in viral maintenance and/or amplification in the enzootic cycle of WNV in the Po valley of Italy, and consequently highlights health impact of WNV and USUV from an ecological point of view in terms of biodiversity loss and harbored threat for some wildlife populations and specifically for the birds of prey as recently discussed by Williams et al. [17].

Evidence of WNV circulation in Columbiformes populations and in Passeriformes (such as corvids) highlights the importance of this kind of surveillance in endemic area since these birds are mainly considered synanthropic and invasive in urban areas increasing the risk of viral transmission to the local human population.

The low detection of other orthoflaviviruses than WNV-1 and WNV-2 during the epidemic season in 2022 (primarily USUV, Marisma virus and Mosquitoes Flavivirus) and the different trend in 2023 suggest a possible biological competition between different viral species of the genus *Orthoflavivirus* according to previous field [13,28] and experimental observations [51].

Unexpected transmission dynamics of several arboviruses are well-known worldwide including Europe and Italy but there is still a lack of knowledge about the epidemiological key aspects of the complex enzootic cycle of this vector-borne disease, capable of explaining with certainty the fluctuations of arboviruses epidemics.

Seasonal transmission dynamics of arboviruses can show different scenarios year by year, suggesting that environmental factors can play a crucial role in the enzootic cycle of these pathogens as well in the viral diversity. Several studies aimed to put in correlation environmental drivers and WNV circulation across Europe and neighbored countries, showing that temperature, precipitation, relative humidity, vegetation cover, presence of wetland areas and land cover can influence the incidence of infection [14,52,53].

On the other hand, environmental factors (biotic and abiotic) are well known to have a positive correlation with viral dispersal and prevalence of European strains of WNV [14,52].

In detail, a drier winter, high spring temperatures and drought during the summer season have been associated with earlier and higher circulation of WNV in vectors, birds and humans in Europe [53], and interestingly analysis of weather conditions of the Veneto Region in the winter and spring–summer seasons in 2022 supported the above-mentioned forecast patterns. Thus, the lack of water supply in the natural environment could enhance the sympatry between mosquitoes and bird populations promoting an increased viral load in the enzootic cycle of WNV [54]. Moreover, the typology of land cover (crop vegetation density, wetland areas for long distance migratory birds), land use (agriculture and livestock) and mammal richness as well as the increased average temperatures due to climate change have been demonstrated to be positively correlated with WNV circulation [14]. The Veneto Region is a plain landscape rich of wetlands, acting as resting and wintering sites across the Eurasian Anatidae flyway [21], and with high agriculture (crops) and livestock farming.

Since the scope of this manuscript is to report descriptively the results of integrated surveillance for WNV and USUV in 2022–2023, future studies will be implemented in order to put in correlation environmental drivers and WNV and/or USUV epidemiology in the Veneto Region exploiting the entomological, veterinary and human datasets developed and described in this study.

The application of the surveillance strategies recommended in the PNA 2020–2025 allowed the early detection of WNV in mosquitoes and/or birds of the Veneto Region before the confirmation of WNV infection in equids and humans during the epidemic in 2022 and 2023. Thus, mosquitoes and birds (especially Passeriformes) are confirmed as the best target for the early detection of WNV and for the implementation of prevention measures in Public Health, including WNV screening in SoHO donors, especially in endemic regions for orthoflavivirus circulation [55,56,57]. The results shown in this study support the efficacy of the One Health approach for WNV surveillance within the Italian Health System, as demonstrated by previous studies [57,58,59,60].

## 6. Conclusions

The present study highlights the importance and the effectiveness of the Italian integrated surveillance plan for WNV, a unique example of a One Health approach since 2008, where human medicine, entomology and veterinary disciplines are interconnected and mutually functional.

## Figures and Tables

**Table 1:** Mosquito species collected in the Veneto Region in 2022 and 2023 during entomological surveillance with CDC-CO_2_ and Gravid traps.

Genus	Species	2022 N (%)	2023 N (%)
*Aedes*	*Ae. albopictus* **	4161 (4.46)	4784 (3.58)
	*Ae. cantans*	19 (0.02)	1 (0.00)
	*Ae. caspius* **	28,094 (30.14)	11,478 (8.59)
	*Ae. detritus*	2 (0.00)	14 (0.01)
	*Ae. geniculatus*	2 (0.00)	5 (0.00)
	*Ae. koreicus*	32 (0.03)	11 (0.01)
	*Ae. vexans*	482 (0.52)	1307 (0.98)
*Anopheles*	*An. claviger/petragnani*	4 (0.00)	5 (0.00)
	*An. maculipennis* s.l.	654 (0.70)	244 (0.18)
	*An. plumbeus*	8 (0.01)	9 (0.01)
*Coquillettidia*	*Cq. richiardii*	39 (0.04)	2 (0.00)
*Culex*	*Cx. hortensis*	0	2 (0.00)
	*Cx. modestus* *	13 (0.01)	40 (0.03)
	*Cx. pipiens* *	54,937 (58.94)	110,800 (82.90)
	*Cx.* spp. *	2 (0.00)	0
*Culiseta*	*Cs. annulata*	6 (0.01)	32 (0.02)
	*Cs.* spp.	1 (0.00)	0
	*Cs. longiareolata*	0	1 (0.00)
Not identified		4757 (5.10)	4913 (3.68)
**Total**		**93,213**	**133,648**

N: number of collected mosquitoes; %: relative abundance. *: species considered main vector or ** potential vector analyzed for orthoflaviviruses.

**Table 2:** WNV and USUV detection in single or co-infections in mosquito pools collected in provinces of the Veneto Region in 2022.

Viruses	Province						Total in the Veneto Region (Tot = 2123) Pos (%)
	Padua (Tot = 269) Pos (%)	Rovigo (Tot = 559) Pos (%)	Treviso (Tot = 207) Pos (%)	Venice (Tot = 572) Pos (%)	Verona (Tot = 336) Pos (%)	Vicenza (Tot = 180) Pos (%)	
WNV-1	12 (4.46)	13 (2.33)	1 (0.48)	8 (1.40)	3 (0.89)	1 (0.56)	**38** (1.79)
WNV-2	1 (0.37)	13 (2.33)	4 (1.93)	18 (3.15)	4 (1.19)	4 (2.22)	**44** (2.07)
WNV-1 and WNV-2	1 (0.37)	3 (0.54)	0	3 (0.52)	0	0	**7** (0.33)
USUV	1 (0.37)	1 (0.18)	0	1 (0.17)	3 (0.89)	2 (1.11)	**8** (0.38)
WNV-1 and USUV	1 (0.37)	0	1 (0.48)	0	0	0	**2** (0.09)
WNV-2 and USUV	0	0	1 (0.48)	1 (0.17)	1 (0.30)	0	**3** (0.14)
WNV-1, WNV-2 and USUV	1 (0.37)	0	0	0	0	0	**1** (0.05)
**Total**	**17** (6.32)	**30** (5.37)	**7** (3.38)	**31** (5.42)	**11** (3.27)	**7** (3.89)	**103** (4.85)

Tot: total pools; Pos: positive pools; %: infection rate.

**Table 3:** WNV and USUV detection in single or co-infections in mosquito pools collected in provinces of the Veneto Region in 2023.

Viruses	Province						Total in the Veneto Region (Tot = 2301) Pos (%)
	Padua (Tot = 366) Pos (%)	Rovigo (Tot = 550) Pos (%)	Treviso (Tot = 258) Pos (%)	Venice (Tot = 557) Pos (%)	Verona (Tot = 375) Pos (%)	Vicenza (Tot = 195) Pos (%)	
WNV-1	0	2 (0.36)	0	2 (0.36)	0	0	**4** (0.17)
WNV-2	5 (1.37)	2 (0.36)	1 (0.39)	5 (0.90)	5 (1.33)	0	**18** (0.78)
WNV-1 and WNV-2	0	0	0	0	0	0	**0**
USUV	2 (0.55)	1 (0.18)	1 (0.39)	6 (1.08)	3 (0.80)	3 (1.54)	**16** (0.70)
WNV-1 and USUV	0	0	0	1 (0.18)	0	0	**1** (0.04)
WNV-2 and USUV	0	1 (0.18)	0	0	1 (0.27)	0	**2** (0.09)
WNV-1, WNV-2 and USUV	0	0	0	0	0	0	**0**
**Total**	**7** (1.91)	**6** (1.09)	**2** (0.78)	**14** (2.51)	**9** (2.40)	**3** (1.54)	**41** (1.78)

Tot: total pools; Pos: positive pools; %: infection rate.

**Table 4:** Total number and relative abundance of birds (grouped by taxonomic order) collected in the Veneto Region by veterinary surveillance in 2022 and 2023.

Order	2022 N (%)	2023 N (%)
Accipitriformes	44 (2.01)	33 (1.82)
Anseriformes	54 (2.46)	44 (2.43)
Apodiformes	100 (4.56)	64 (3.53)
Bucerotiformes	1 (0.05)	1 (0.06)
Caprimulgiformes	1 (0.05)	0
Charadriiformes	161 (7.34)	181 (9.99)
Columbiformes	401 (18.29)	441 (24.34)
Coraciiformes	3 (0.14)	8 (0.44)
Falconiformes	93 (4.24)	50 (2.76)
Galliformes	27 (1.23)	11 (0.61)
Gaviiformes	0	1 (0.06)
Gruiformes	11 (0.50)	12 (0.66)
Passeriformes	942 (42.95)	701 (38.69)
Pelecaniformes	62 (2.83)	47 (2.59)
Phoenicopteriformes	2 (0.09)	0
Piciformes	53 (2.42)	21 (1.16)
Podicipediformes	3 (0.14)	0
Psittaciformes	0	7 (0.39)
Strigiformes	182 (8.30)	124 (6.84)
Not identified	53 (2.42)	66 (3.64)
**Total**	**2193**	**1812**

N: number of birds collected; %: relative abundance.

**Table 5:** Avifauna positive for WNV-1, WNV-2, and USUV in single or co-infections during the veterinary surveillance in 2022.

Order	Common Name	Scientific Name	MP	WNV-1 Pos/N (%)	WNV-2 Pos/N (%)	WNV-1 and WNV-2 Pos/N (%)	USUV Pos/N (%)
Accipitriformes	Eurasian Sparrowhawk	*Accipiter nisus*	M ^†^	1/27 (3.70)	0/27	0/27	0/27
Anseriformes	Mallard	*Anas platyrhynchos*	M	1/17 (5.88)	0/17	0/17	1/17 (5.88)
Apodiformes	Common Swift	*Apus apus*	M	5/100 (5.00)	0/100	0/100	0/100
Charadriiformes	European Herring Gull	*Larus argentatus*	M ^†^	2/105 (1.90)	0/105	0/105	0/105
	Seagull	*Larus* spp.	M ^†^	5/32 (15.63)	3/32 (9.38)	0/32	0/32
Columbiformes	Common Wood Pigeon	*Columba palumbus*	M ^†^	6/99 (6.06)	3/99 (3.03)	1/99 (1.01)	2/99 (2.02) +1 *
	European Turtle Dove	*Streptopelia* spp.	M	3/180 (1.67)	4/180 (2.22)	0/180	0/180
	Rock Dove	*Columba livia*	R	1/122 (0.82)	0/122	0/122	0/122
Falconiformes	Common Kestrel	*Falco tinnunculus*	M ^†^	4/88 (4.55)	4/88 (4.55)	1/88 (1.14)	0/88
Galliformes	Pheasant	*Phasianus colchicus*	R	1/12 (8.33)	0/12	0/12	0/12
Passeriformes	Barn Swallow	*Hirundo rustica*	M	1/33 (3.03)	1/33 (3.03)	0/33	0/33
	Carrion Crow	*Corvus corone*	R	14/133 (10.53)	6/133 (4.51)	1/133 (0.75)	0/133
	Common Blackbird	*Turdus merula*	M ^†^	12/250 (4.80)	3/250 (1.20)	0/250	9/250 (3.60)
	Domestic Canary	*Serinus canaria domestica*	R	1/7 (14.29)	1/7 (14.29)	0/7	0/7
	Eurasian Jay	*Garrulus glandarius*	M ^†^	4/34 (11.76)	1/34 (2.94)	1/34 (2.94)	0/34
	Eurasian Magpie	*Pica pica*	R	8/286 (2.80)	8/286 (2.80)	0/286	1/286 (0.35)
	European Goldfinch	*Carduelis carduelis*	M ^†^	1/10 (10.00)	0/10	0/10	0/10
	Rook	*Corvus* spp.	M ^†^	1/2 (50.00)	0/2	0/2	0/2
	Sparrow	*Passer* spp.	R	4/35 (11.43)	2/35 (5.71)	0/35	1/35 (2.86)
Pelecaniformes	Great Cormorant	*Phalacrocorax carbo*	R	1/5 (20.00)	1/5 (20.00)	0/5	0/5
	Grey Heron	*Ardea cinerea*	M ^†^	1/16 (6.25)	0/16	0/16	0/16
	Little Egret	*Egretta garzetta*	M	1/12 (8.33)	0/12	0/12	0/12
	Little Bittern	*Ixobrychus minutus*	M	2/5 (40.00)	0/5	0/5	0/5
	Purple Heron	*Ardea purpurea*	M	0/3	1/3 (33.33)	0/3	0/3
	Western Cattle Egret	*Bubulcus ibis*	M ^†^	2/18 (11.11)	1/18 (5.56)	0/18	1/18 (5.56)
Piciformes	European Green Woodpecker	*Picus viridis*	R	2/13 (15.38)	0/13	0/13	0/13
	Woodpecker	n.a.	R	1/34 (2.94)	0/34	0/34	0/34
Strigiformes	Eurasian Scops Owl	*Otus scops*	R	2/28 (7.14)	5/28 (17.86)	0/28	0/28
	Little Owl	*Athene noctua*	R	21/120 (17.50)	2/120 (1.67)	3/120 (2.50)	0/120
	Long-Eared Owl	*Asio otus*	M ^†^	1/19 (5.26)	1/19 (5.26)	0/19	0/19
	Tawny Owl	*Strix aluco*	R	0/4	0/4	0/4	1/4 (25.00)
	Western Barn Owl	*Tyto alba*	R	2/11 (18.18)	0/11	0/11	0/11
**Infection Rate (Tot = 2193)**				**111** (5.06)	**47** (2.14)	**7** (0.32)	**16** (0.73) +**1** *

Pos: positive birds; N: number of birds collected; %: infection rate; MP: migration pattern according to Avibase [40]; M: migrant; ^†^: birds with a partial or total migration attitude; R: resident; n.a.: not applicable; *: bird positive for USUV and WNV-1; Tot: total birds collected in 2022.

**Table 6:** Avifauna positive for WNV-1, WNV-2 and/or USUV in single or co-infections during the veterinary surveillance in 2023.

Order	Common Name	Scientific Name	MP	WNV-1 Pos/N (%)	WNV-2 Pos/N (%)	USUV Pos/N (%)
Anseriformes	Gadwall	*Mareca strepera*	M	0/2	0/2	0/2 +1 *
	Mallard	*Anas platyrhynchos*	M	0/22	1/22 (4.55)	1/22 (4.55)
Charadriiformes	European Herring Gull	*Larus argentatus*	M ^†^	1/106 (0.94)	0/106	0/106
	Seagull	*Larus* spp.	M ^†^	0/20	1/20 (5.00)	0/20
Columbiformes	Common Wood Pigeon	*Columba palumbus*	M ^†^	4/144 (2.78)	6/144 (4.17)	5/144 (3.47) +1 * +8 **
	European Turtle Dove	*Streptopelia* spp.	M	3/163 (1.84)	3/163 (1.84)	3/163 (1.84) +1 **
	Rock Dove	*Columba livia*	R	0/134	2/134 (1.49)	1/134 (0.75)
Falconiformes	Common Kestrel	*Falco tinnunculus*	M ^†^	0/44	1/44 (2.27)	0/44
Passeriformes	Barn Swallow	*Hirundo rustica*	M	0/19	1/19 (5.26)	0/19
	Carrion Crow	*Corvus corone*	R	2/131 (1.53)	2/131 (1.53)	0/131
	Common Blackbird	*Turdus merula*	M ^†^	0/138	0/138	10/138 (7.25) +1 *
	Common House Martin	*Delichon urbicum*	M	0/15	2/15 (13.33)	0/15
	Eurasian Jay	*Garrulus glandarius*	M ^†^	3/36 (8.33)	2/36 (5.56)	0/36
	Eurasian Magpie	*Pica pica*	R	1/247 (0.40)	4/247 (1.62)	0/247
	European Greenfinch	*Chloris chloris*	M ^†^	0/3	1/3 (33.33)	0/3
	Hooded Crow	*Corvus cornix*	R	1/2 (50.00)	0/2	0/2
	House Sparrow	*Passer domesticus*	R	0/2	1/2(50.00)	0/2
	Sparrow	*Passer* spp.	R	0/9	0/9	1/9 (11.11)
Pelecaniformes	Black-Crowned Night-Heron	*Nycticorax nycticorax*	M	0/2	0/2	1/2 (50.00)
	Grey Heron	*Ardea cinerea*	M ^†^	0/15	0/15	0/15 +1 *
	Little Egret	*Egretta garzetta*	M	0/6	1/6 (16.67)	0/6
	Purple Heron	*Ardea purpurea*	M	0/1	0/1	1/1 (100)
	Western Cattle Egret	*Bubulcus ibis*	M ^†^	0/10	1/10 (10.00)	0/10
Psittaciformes	Parrot	n.a.	R	1/7 (14.29)	0/7	0/7
Strigiformes	Eurasian Scops Owl	*Otus scops*	R	0/22	1/22 (4.55)	1/22 (4.55)
	Little Owl	*Athene noctua*	R	0/85	2/85 (2.35)	0/85
**Infection Rate (Tot = 1812)**				**16** (0.89)	**32** (1.79)	**24** (1.34) +**4** * +**9** **

Pos: positive birds; N: number of birds collected; %: infection rate; MP: migration pattern according to Avibase [40]; M: migrant; ^†^: birds with a partial or total migration attitude; R: resident; *: birds positive for USUV and WNV-1; **: birds positive for USUV and WNV-2; Tot: total birds collected in 2023; n.a.: not applicable.

**Table 7:** Distribution of confirmed human infections by the province of patients’ residence in Veneto during 2022 and 2023.

Year	Province	WNV *	WNV-1	WNV-2	Total (%)
2022	Belluno **	0	2	0	**2** (0.38)
	Padua	114	126	38	**278** (52.35)
	Rovigo	11	17	8	**36** (6.78)
	Treviso	23	7	9	**39** (7.34)
	Venice	44	26	21	**91** (17.14)
	Verona	19	5	14	**38** (7.16)
	Vicenza	21	20	6	**47** (8.85)
**Total** (%)		**232** (43.69)	**203** (39.23)	**96** (18.08)	**531**
2023	Padua	9	9	6	**24** (42.86)
	Rovigo	1	1	0	**2** (3.57)
	Treviso	1	0	0	**1** (1.79)
	Venice	2	4	2	**8** (14.29)
	Verona	6	0	11	**17** (30.36)
	Vicenza	4	0	0	**4** (7.14)
**Total** (%)		**23** (41.07)	**14** (25.00)	**19** (33.93)	**56**

%: relative abundance. * Human infections where the lineage of WNV were not determined. ** Belluno Province: this province is considered at low risk for orthoflavivirus circulation, thus entomological surveillance and active surveillance in bird target species are not planned (as reported on PNA 2020–2025). Patient exposure likely occurred while outside the Belluno Province.

## Data Availability

The original contributions presented in this study are included in the article/Supplementary Material. Further inquiries can be directed to the corresponding author.

## Supplementary Materials

The following supporting information can be downloaded at: https://www.mdpi.com/article/10.3390/pathogens14030227/s1, Figure S1: Temporal distribution of positive mosquito pools for orthoflaviviruses in provinces of the Veneto Region in 2022; Figure S2: Temporal distribution of positive mosquito pools for orthoflaviviruses in provinces of the Veneto Region in 2023; Figure S3: Temporal distribution of avifauna positive samples for orthoflaviviruses in provinces of the Veneto Region in 2022; Figure S4: Temporal distribution of avifauna positive samples for orthoflaviviruses in provinces of the Veneto Region in 2023; Table S1: Mosquitoes traps 2022–2023; Table S2a: Orthoflaviviruses detection in mosquito pools collected in provinces of the Veneto Region in 2022; Table S2b: Orthoflaviviruses detection in mosquito pools collected in provinces of the Veneto Region in 2023; Table S3a: Statistical analysis of the infection rate in bird orders collected between the years 2022 and 2023; Table S3b: Statistical analysis of the relationship between positive birds and migratory patterns; Table S3c: Statistical analysis of distribution of human cases by virus types observed between 2022 and 2023.
