# Supplementary figures and images for: Integrated One Health Surveillance of West Nile Virus and Usutu Virus in the Veneto Region, Northeastern Italy, from 2022 to 2023
Source: Pathogens. 2025 Feb 25;14(3):227. doi: 10.3390/pathogens14030227 (PMC11945005; doi:10.3390/pathogens14030227)

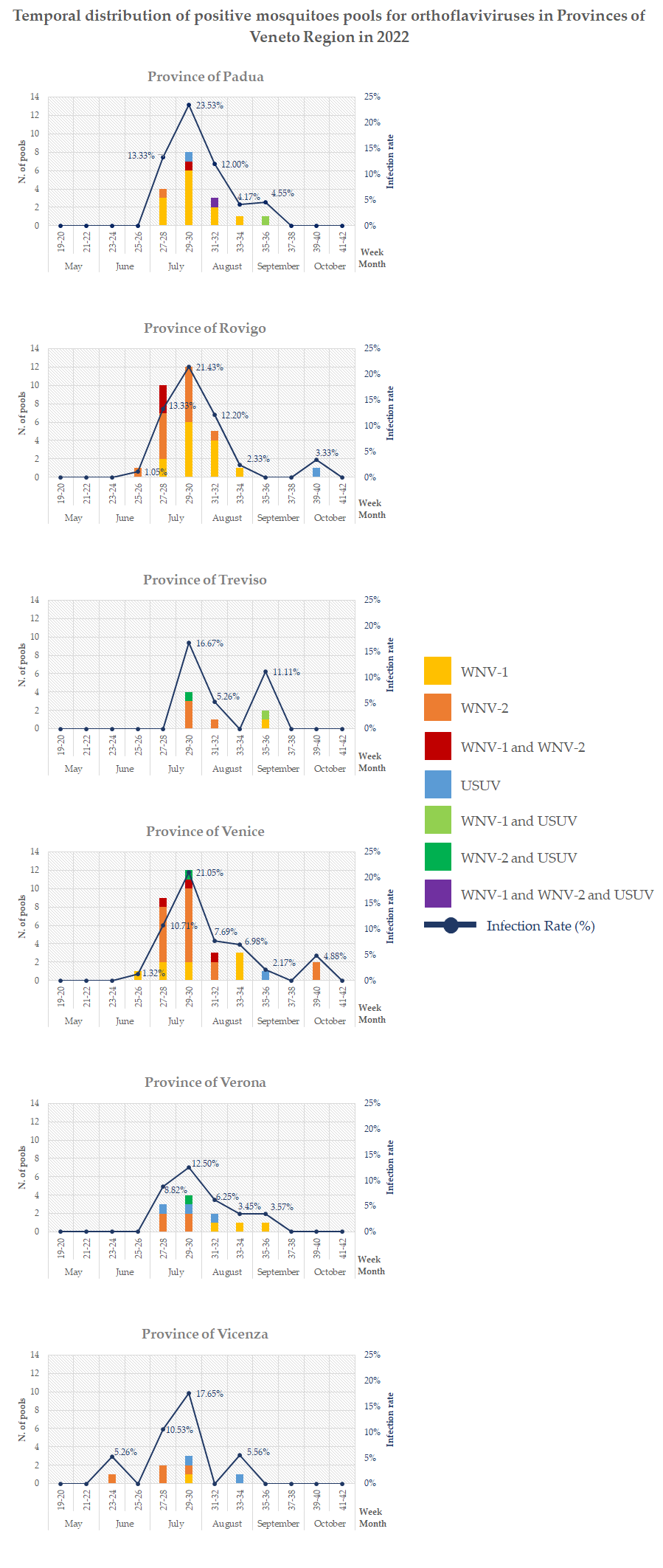

Supplement: Supplementary file 1 [file pathogens-14-00227-s001.zip › Figure S1 Mos 2022 temporal trend in Provinces.png]

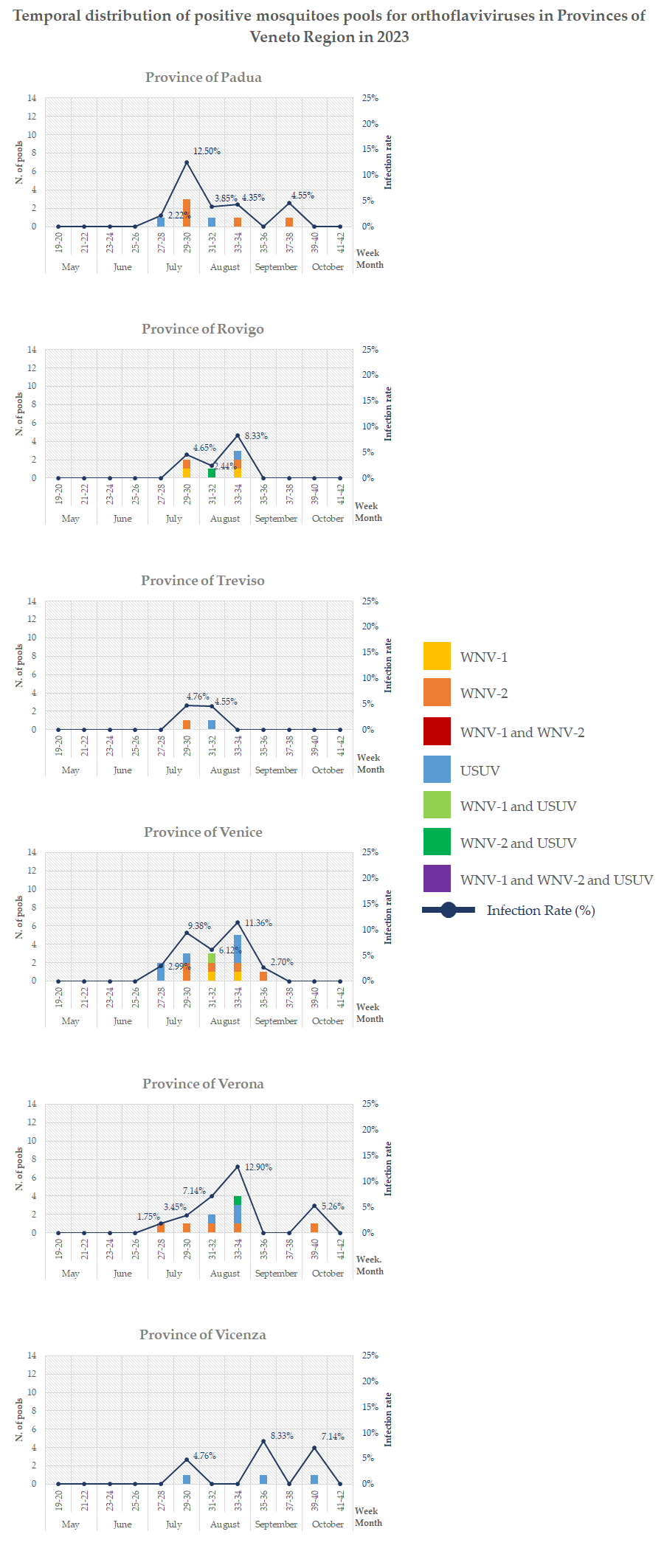

Supplement: Supplementary file 1 [file pathogens-14-00227-s001.zip › Figure S2 Mos 2023 temporal trend in Provinces.png]

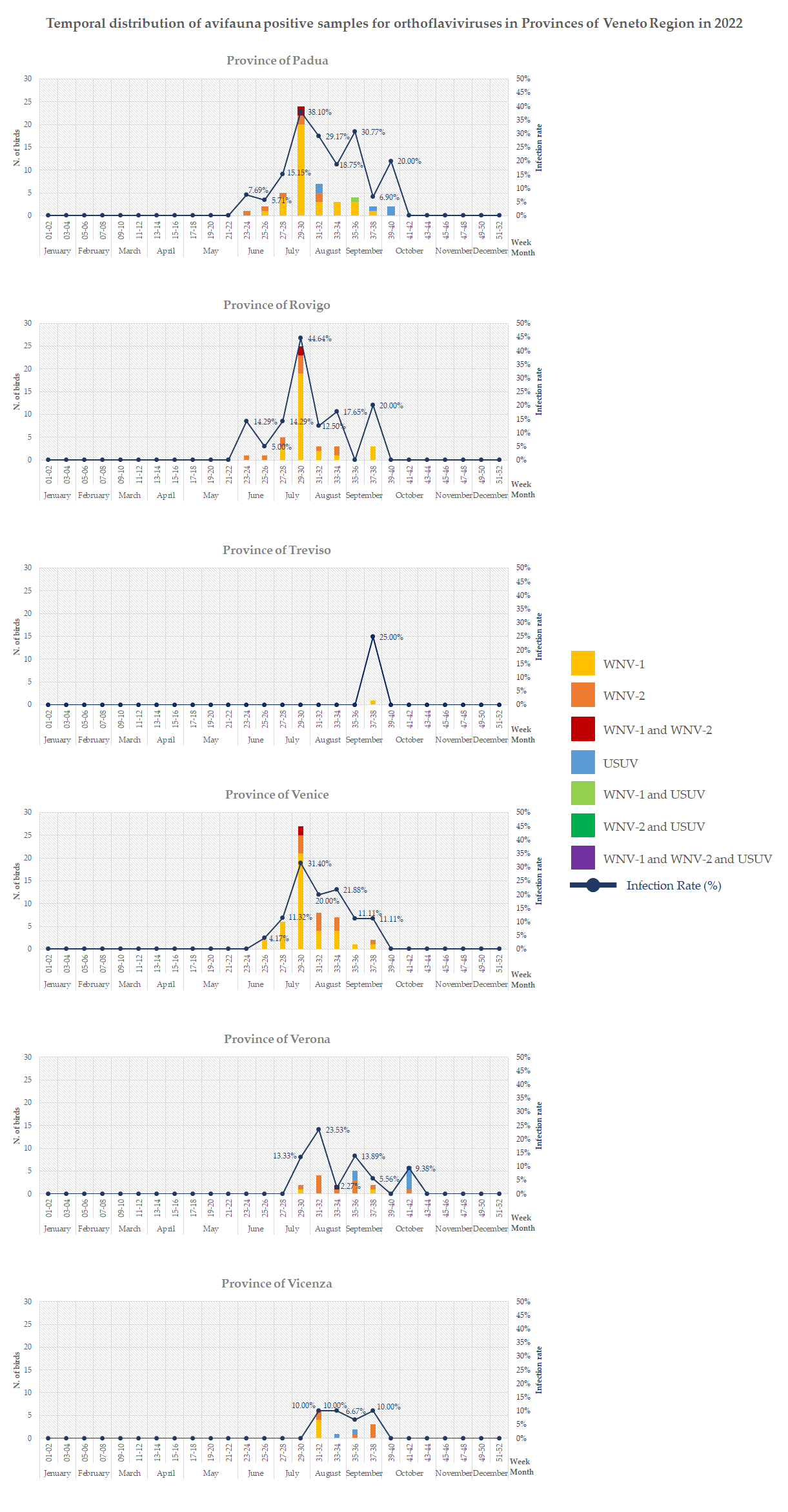

Supplement: Supplementary file 1 [file pathogens-14-00227-s001.zip › Figure S3 Birds 2022 temporal trend in Provinces.png]

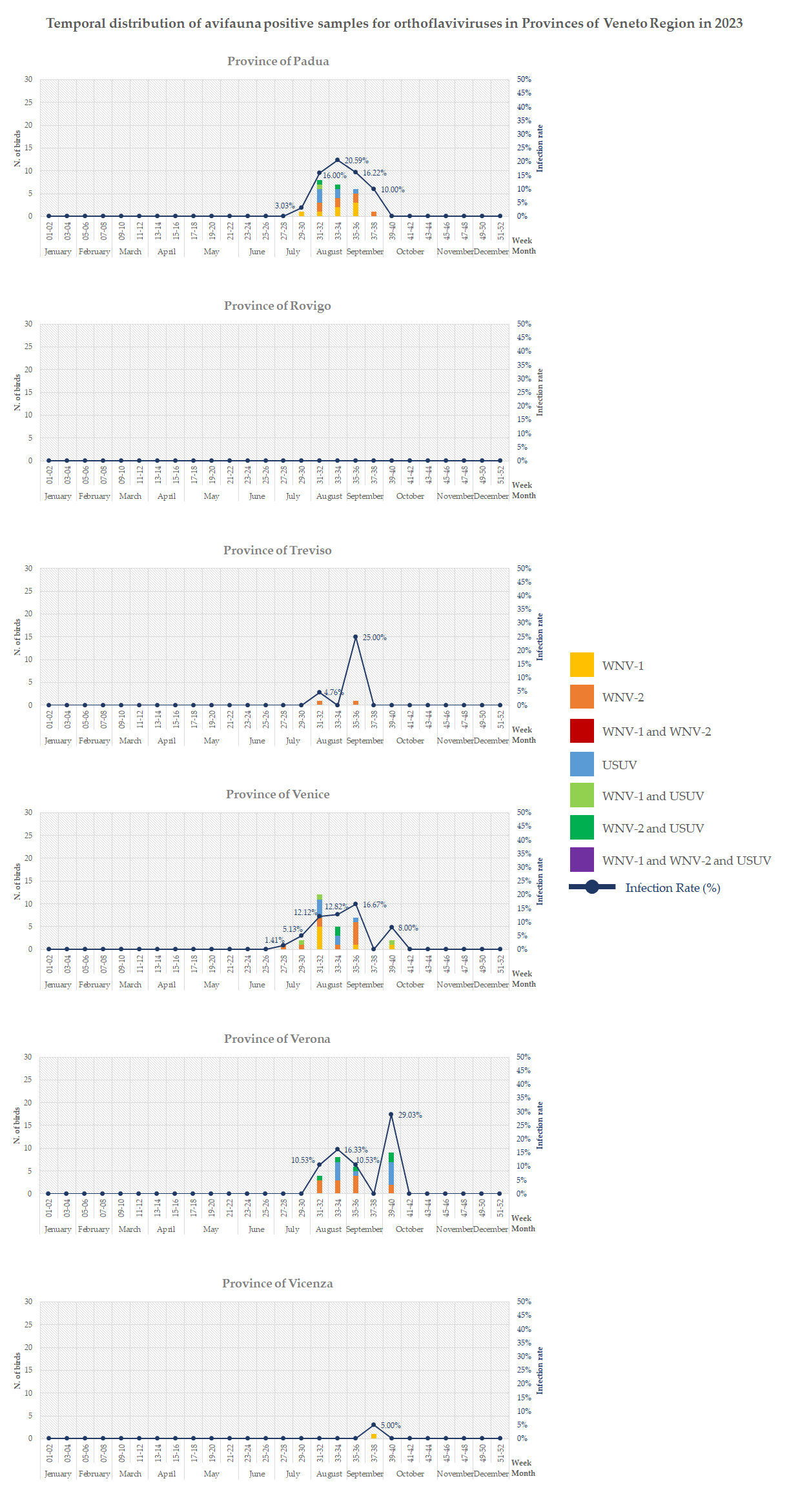

Supplement: Supplementary file 1 [file pathogens-14-00227-s001.zip › Figure S4 Birds 2023 temporal trend in Provinces.png]
